# Liver Transcriptome and Gut Microbiome Analysis Reveals the Effects of High Fructose Corn Syrup in Mice

**DOI:** 10.3389/fnut.2022.921758

**Published:** 2022-06-30

**Authors:** Yu Shen, Yangying Sun, Xiaoli Wang, Yingping Xiao, Lingyan Ma, Wentao Lyu, Zibin Zheng, Wen Wang, Jinjun Li

**Affiliations:** ^1^State Key Laboratory for Managing Biotic and Chemical Threats to the Quality and Safety of Agro-products, College of Food and Pharmaceutical Sciences, Ningbo University, Ningbo, China; ^2^Institute of Agro-product Safety and Nutrition, Zhejiang Academy of Agricultural Sciences, Hangzhou, China; ^3^Institute of Food Sciences, Zhejiang Academy of Agricultural Sciences, Hangzhou, China

**Keywords:** gut microbiota, hepatic lipid metabolism, high fructose corn syrup, transcriptome analysis, mouse

## Abstract

High fructose corn syrup (HFCS) is a viscous mixture of glucose and fructose that is used primarily as a food additive. This article explored the effect of HFCS on lipid metabolism-expressed genes and the mouse gut microbiome. In total, ten 3-week-old male C57BL/6J mice were randomly divided into two groups, including the control group, given purified water (Group C) and 30% HFCS in water (Group H) for 16 weeks. Liver and colonic content were collected for transcriptome sequencing and 16S rRNA gene sequencing, respectively. HFCS significantly increased body weight, epididymal, perirenal fat weight in mice (*p* < 0.05), and the proportion of lipid droplets in liver tissue. The expression of the ELOVL fatty acid elongase 3 (Elovl3) gene *was* reduced, while Stearoyl-Coenzyme A desaturase 1 (*Scd1*), peroxisome proliferator activated receptor gamma (*Pparg)*, fatty acid desaturase 2 (*Fads2*), acyl-CoA thioesterase 2 (*Acot2*), acyl-CoA thioesterase 2 (*Acot3*), acyl-CoA thioesterase 4 (*Acot4*), and fatty acid binding protein 2 (*Fabp2*) was increased in Group H. Compared with Group C, the abundance of Firmicutes was decreased in Group H, while the abundance of Bacteroidetes was increased, and the ratio of Firmicutes/Bacteroidetes was obviously decreased. At the genus level, the relative abundance of *Bifidobacterium, Lactobacillus, Faecalibaculum, Erysipelatoclostridium*, and *Parasutterella* was increased in Group H, whereas that of *Staphylococcus, Peptococcus, Parabacteroides, Donghicola*, and *Turicibacter was* reduced in Group H. *Pparg, Acot2, Acot3*, and *Scd1* were positively correlated with *Erysipelatoclostridium* and negatively correlated with *Parabacteroides, Staphylococcus*, and *Turicibacter*. *Bifidobacterium* was negatively correlated with *Elovl3*. Overall, HFCS affects body lipid metabolism by affecting the expression of lipid metabolism genes in the liver through the gut microbiome.

## Introduction

High fructose corn syrup (HFCS) is a viscous mixture of glucose and fructose. In recent years, due to the rising price of sugar, the low price and better taste of HFCS have been favored by the food industry, especially in beverage production, and it is also a key ingredient in baked goods, desserts, and juices (1). Fructose in the diet may contribute to increased energy intake and weight gain, which can lead to problems, such as obesity (2). Furthermore, an HFCS diet significantly alters the community structure of the gut microbiome and lipid metabolism (3, 4).

The liver is primarily the site of fructose metabolism, which is an essential organ in the body related to lipid metabolism (5, 6) and plays an important role in regulating appetite and body weight (7). Li et al. found that most of the changes in the liver transcriptome level of laying hens are closely related to fat metabolism by performing transcriptome analysis on the liver of young and laying hens (8). It was shown that peroxisome proliferator activated receptor gamma (*Pparg*) is a key factor in lipid formation and can be combined with specific substances to reduce lipid formation (9). Studies on broilers of the same breed and different body weights showed that the expression of *LIPG* and *CPT1A* genes is related to lipid metabolism (10, 11). Emmanuelle (12) adipose regulation in animals of different body sizes is related to the regulation of genes involved in adipogenesis. Furthermore, the expression of different genes in the organism may lead to differences in fat deposition (13).

The gut microbiome affects host lipid metabolism and plays an important role in the development of metabolic diseases through interaction with the diet (14, 15). The human gut contains a diverse and complex microbial community that plays an important role in energy and lipid metabolism (16). The gut microbiome is colonized by 10^14^ macroorganisms of numerous species, which are important components of the human gut microbiome ecosystem (17). The microbiome in the gastrointestinal tract of mice consists mainly of Firmicutes (74%) and Bacteroidetes (23%) at the phylum level (18). A high-fructose diet altered the Firmicutes/Bacteroidetes ratio and the abundance of *Parasutterella, Lactobacillus, Bifidobacteriaceae*, and *Alistipes* (19, 20). Crescenzo et al. (21) indicated that a fructose diet alters the mouse gut microbiome, which contributes to the dysregulation of liver metabolism.

Thus, to better understand the effects of HFCS intake on mouse metabolism, this study is aimed to determine the effects of HFCS intake on the gene expression of liver lipid metabolism and the community structure and function of the gut microbiome in mice and the association between the gene expression and the gut microbiome.

## Materials and Methods

### Ethics Statement

The study was conducted at the Laboratory Animal Center of Zhejiang Academy of Agricultural Sciences (Animal Experimentation License No. 286868667) and was approved by the Zhejiang Provincial Ethics Committee for Laboratory Animals (Ethical Approval No. 78865576). All methods were conducted following the relevant guidelines and regulations.

### Preparation of Animal and Liver Tissue Samples

Male C57BL/6J mice of 3-week-old were purchased from Shanghai Slack Company. After all mice were adaptively fed for 1 week, they were randomly divided into two groups, the control group (Group C) and the HFCS group (Group H), which were fed drinking water containing 30% HFCS. All mice were fed at room temperature and relative humidity of 50–70%. Each group was exposed to light for 12 h a day, and the mice were free to eat and drink. After 16 weeks of intervention, water was removed without fasting for 12 h. The mice were weighed, and the liver, epididymal fat, and perirenal fat were quickly weighed. To analyze the liver, the left leaf was placed in 4% paraformaldehyde, and the right leaf was stored at −80°C. Data were recorded in time for all operations. The liver samples for examination were fixed with 4% paraformaldehyde. After the fixed state was achieved, the samples were pruned, dehydrated, embedded, sectioned, stained, and sealed in strict accordance with the standard operating procedure (SOP) of the unit for pathological experiment detection and finally qualified for microscopic examination. The whole intestine was separated from the abdominal cavity of the mice, and the content of the colon was removed and stored on dry ice.

### RNA Extraction

Total RNA was isolated from the liver tissues of 10 mice. TRIzol (Invitrogen, USA) was used to purify the extract, and the purity of RNA (ratio of OD260/280 and OD260/230) was detected by a Nano Spectrophotometer (Implen, Maryland, CA, USA). An Agilent 2100 Bioanalyzer (Agilent Technologies, Santa Clara, CA, USA) was used to accurately detect the integrity and total amount of RNA.

### Library Construction and Sequencing

According to the manufacturer's suggestion, the kit used to build the library was the NEBNext^®^ Ultra™ RNA Library Prep Kit for illumination. After the library was built, Qubit 2.0 Fluorometer was used for preliminary quantification, and the library was diluted to 1.5 ng/μl. Then, an Agilent 2100 Bioanalyzer was used to detect the insert size of the library. After the insert size met the expectation, quantitative real-time PCR (qRT-PCR) accurately quantified the effective concentration of the library (the effective concentration of the library was higher than 2 nM) to ensure the quality of the library. After library inspection was qualified, different libraries were pooled according to the requirements of effective concentration and target off-board data volume. Then, Illumina sequencing was performed, and a 150 bp paired-end reading was generated.

### Differential Gene Expression Analysis

DESeq2 software (1.20.0) was used to analyze the differential expression between the two comparison combinations. DESeq2 provides statistical procedures for determining differential expression in digital gene expression data using a model based on a negative binomial distribution. Benjamini and Hochberg's method was used to adjust the *p*-value to control the error detection rate. Genes with an adjusted *p* < 0.05 found by DESeq2 were called differentially expressed genes (DEGs).

### DEGs of Gene Oncology (GO) and Kyoto Encyclopedia Gene and Genome (KEGG) Enrichment Analyses

Gene Oncology enrichment analysis of DEGs was realized by Cluster Profiler (3.4.4) software, in which the gene length bias was corrected, and GO terms with corrected *p*-values <0.05 were significantly enriched by DEGs. We used Cluster Profiler (3.4.4) software analysis of DEGs in KEGG pathway enrichment after using a local version of the GSEA tool http://www.broadinstitute.org/gsea/index. jsp, GO to the species, KEGG GSEA datasets. The KEGG (http://www.genome.jp/KEGG/) (22) information from the molecular level, especially the genome sequencing high flux experimental technology of large-scale molecular datasets, understands cells, biological, ecological system, the advanced features of biological systems, and the utility of the database resources. In this study, KOBAS (23) software was used to test the statistical enrichment of DEGs in the KEGG pathway.

### RT-QPCR Analysis

To confirm the reproducibility and accuracy of the RNA sequencing (RNA-Seq) gene expression data obtained from mouse liver libraries, RT-qPCR analysis was performed. The RT-qPCR system (20 μl) was as follows: Power SYBR^®^ Green Master Mix, 10 μl; gene-specific upstream and downstream primers (10 μmol/L), 0.5 μl; sterile water, 8 μl; and cDNA template, 1 μl. The reaction conditions were as follows: 95°C for 1 min, followed by 40 cycles of 95°C for 15 s and 63°C for 25 s (for collecting fluorescence data). The relative expression level of each gene was determined by the 2^−*Ct*^ method using GAPDH as the internal reference gene, and the reaction was repeated three times for each sample. The primers used for quantification in the study were designed using the Primer-Basic Local Alignment Search Tool (BLAST) on the NCBI website (https://www.ncbi.nlm.nih.gov/tools/primer-blast/). Gene information for RT-PCR is shown in Table 1.

**Table 1:** Primers for RT-qPCR.

**Gene**	**Size (bp)**	**Annealing (**°**C)**	**Forward (5'to3')**	**Reword (5'to3')**
*GAPDH*	127	60	GAAGGTCGGTGTGAACGGATTTG	CATGTAGACCATGTAGTTGAGGTCA
*Scd1*	110	60	GCAAGCTCTACACCTGCCTCTTC	CAGCCGTGCCTTGTAAGTTCTG
*Pparg*	133	60	CCAAGAATACCAAAGTGCGATCA	CCCACAGACTCGGCACTCAAT
*Elovl3*	91	60	GTAAGCGTCCACTCATCTTTGTC	CCCGAAGGCACTTTGTTCTTGTAT
*Fads2*	86	60	CGACATTTCCAACACCATGCCAA	CACTCGCCAAGGACAAACAC
*Acot2*	97	60	GACAGGGTTTCTCTGTGTACC	GTGGCTTTACTCCCAGCACTT
*Acot3*	121	60	CTGCTACATCCCTGGAGTTC	CCCTTAACTGCTGAGCCATCTTT
*Acot4*	178	60	GCCTGTAACAGACATGGTAGATTC	CTGTAACAAGCACAGGCTGGTA
*Fabp2*	73	60	CTGATTGCTGTCCGAGAGGTT	GCTTGGCCTCAACTCCTTCATAT

### Histological Staining

The histological morphology of the liver was observed using hematoxylin-eosin staining. After fixation with 4% paraformaldehyde and in good condition, the samples were pruned, dehydrated, embedded, sectioned, stained, and sealed in strict accordance with the procedures of the unit's pathological experiment detection SOP and finally qualified for microscopic examination. Steatosis of liver tissue in mice treated with 30% HFCS was observed.

### DNA Extraction and Sequencing

According to the instructions of the manufacturer, microbial genomic DNA was obtained from each intestinal content (QIAamp DNA Stool Mini Kit QIAGEN, CA, USA). The barcode-specific primers 515 F 5′-barcode-GTGCCAGCMGCCGCGG-3′ and 907 R 5′-CCGTCAATTCMTTTRAGTTT-3′ were synthesized to amplify the V4+V5 region of 16S rRNA gene sequencing (24). The PCRs were performed in triplicate using a 20 μl mixture that contained 4 μl of 5 × FastPfu Buffer, 2 μl of 2.5 mM deoxynucleotide triphosphates (dNTPs), 0.8 μl of forward primer (5 μM), 0.8 μl of reverse primer (5 μM), 0.4 μl of FastPfu Polymerase, 0.2 μl of bovine serum albumin (BSA), 10 ng of template DNA, and ddH_2_O was added to 20 μl. Amplicons were extracted from 2% agarose gels and purified using the AxyPrep DNA Gel Extraction Kit (Axygen Biosciences, Union City, CA, USA) according to the manufacturer's instructions and quantified using a QuantiFluorTM-ST (Promega, USA) Bioinformatics Analysis Illumina paired reads that were multiplexed, and clean reads were screened in the Quantitative Analysis of Microbial Ecology (QIIME) quality filter (25), combined into labels using FLASH (26), and then allocated a unique barcode to each sample. After removing redundancy, the labels of each sample were analyzed, and UPARSE and UCHIME were used to assign unique labels with sequence similarity ≥97% to the same operational taxonomic unit (OTU). Selected OTUs were annotated with taxonomic information using the Ribosomal Database Project (RDP) classifier (27). Alpha diversity and beta diversity were calculated and visualized in GraphPad Prism 8.0.2 (San Diego, CA, USA).

### Statistical Analysis

All the data were statistically analyzed by SPSS version 23 software using one-way ANOVA. The mean ± standard deviation (SD; *x* ± *s*) is indicated, and *p* < 0.05 was considered statistically significant.

## Results

### Body Weight and Fat Deposition in Mice

**Figure 1:**
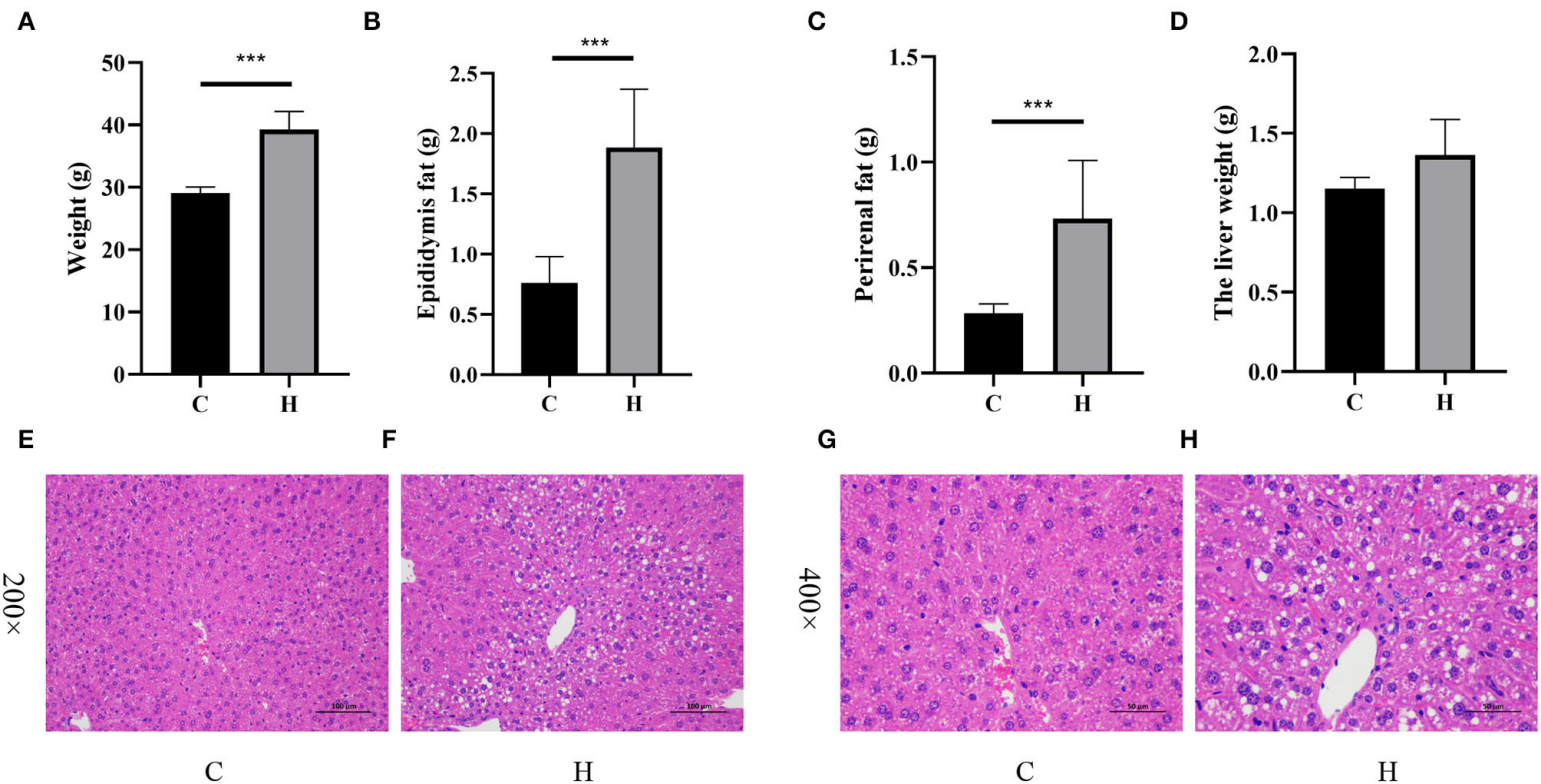
High fructose corn syrup (HFCS) treatment affects body weight and fat deposition in mice. **(A)** Body weight of each group during the intervention period; **(B)** epididymal fat content in each group during the intervention period; **(C)** perirenal fat content in each group during the intervention period; **(D)** liver weight in each group during the intervention period; **(E)** the liver section was enlarged to 200× in Group C; and **(F)** the liver section was enlarged to 200× in Group H. **(G)** The liver section was enlarged to 400× in Group C and **(H)** the liver section was enlarged to 400× in Group H. ***p < 0.001.

The average body weight of mice in Group C was 29.060 ± 0.446 g and that in Group H was 38.220 ± 1.515 g, and the difference between the two groups was extremely significant (*p* < 0.001; Figure 1A). The average weight of epididymal fat showed a significant increase in the H group, which was 1.886 ± 0.216 g, while the weight of epididymal fat in Group C was 0.762 ± 0.098 g (*p* < 0.001; Figure 1B). By comparing the perirenal fat of mice in the two groups, we found that the difference between the two groups was extremely significant, 0.284 ± 0.020 g in Group C and 0.732 ± 0.123 g in Group H (*p* < 0.001; Figure 1C). The results showed that the liver weight of mice in Group H was increased but not significantly different as compared to Group C, while the average liver weight of Group C was 1.152 ± 0.031 g and that of Group H was 1.364 ± 0.100 g (*p* = 0.0778; Figure 1D). Round vacuoles of varying sizes were observed in the cytoplasm, and larger areas of lipid droplets were observed in Group H than in Group C. As shown in Figures 1E–H, it was clear that the liver fat content in Group H was higher than that in Group C.

### Differential Gene Expression Analysis

The Illumina HiSeq library was sequenced using the Illumina HiSeq platform to produce a paired-end reading of 150 bp. All sequencing data have been submitted to the NCBI Comprehensive Gene Expression Database with the registration number PRJNA731593. RNA from 10 samples was used, and the quality control method was mainly used to accurately detect RNA integrity with an Agilent 2100 Bioanalyzer. The results showed that the concentration and total amount of 10 samples met the requirements of sequencing. Ten samples were sequenced using the Hiseq 2500 high-throughput sequencing platform, and the average original reading of the 10 samples was 45,601,720. After removing the sequencing fitting and primer sequences, filtering the low-quality value data, and sequencing quality control, a total of 66.64 GB of clean data were obtained, and the average clean reading of the 10 samples was 44,435,441. In the clean data, Q30 is the percentage of the base with a recognition accuracy of over 99.9%; the reading proportion of the mass value over 30 (Q30) ranges from 94.13 to 94.8%; in the library, the content of gas chromatography ranges from 48.44 to 50.41% (Table 2), indicating good sequencing results.

**Table 2:** Summary of the original data, clean data, sequencing error rate, clean read rate, and clean Q30 and Q20 base rate of the ordered samples.

**Sample**	**raw_reads**	**clean_reads**	**clean_bases**	**Clean Q30** **bases rate (%)**	**Mapped** **ratio (%)**
C1	47,303,062	46,303,274	6.95G	94.80	94.06
C2	47,089,510	46,136,604	6.92G	94.13	93.48
C3	44,272,672	43,279,174	6.49G	94.43	93.85
C4	41,283,960	40,213,348	6.03G	94.40	92.99
C5	46,466,272	45,560,390	6.83G	94.69	93.80
H1	45,602,948	44,095,038	6.61G	94.75	93.34
H2	47,022,136	45,853,632	6.88G	94.56	95.24
H3	46,125,854	44,628,824	6.69G	94.67	93.72
H4	47,664,476	46,361,598	6.95G	94.62	95.05
H5	43,186,314	41,922,528	6.29G	94.67	93.84

**Figure 2:**
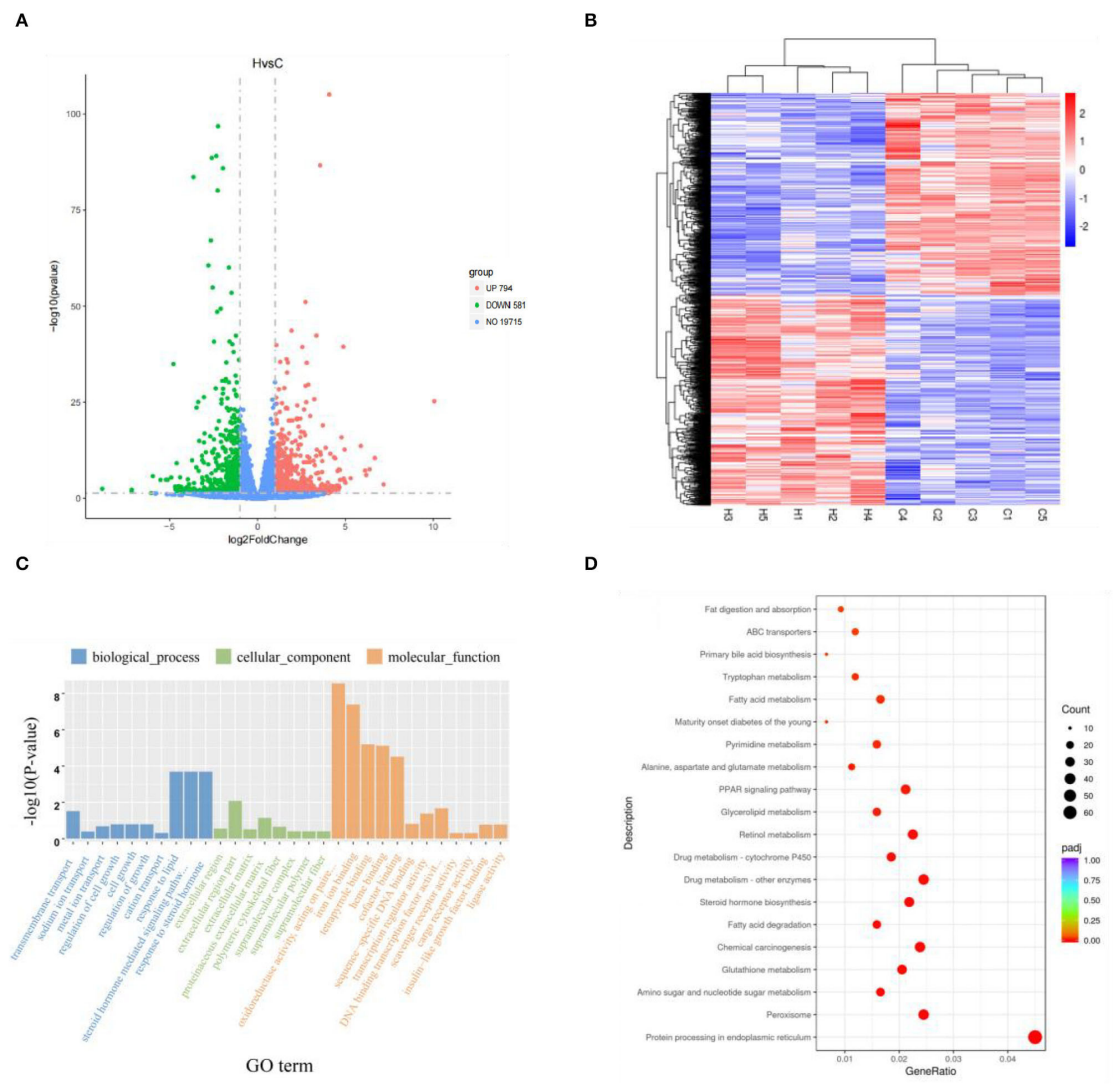
Volcano map of total gene expression of Groups C and H. Red dots represent upregulated genes, green dots represent downregulated genes, and blue dots represent non-differentially expressed genes **(A)**. Expression profiles of 1,375 differentially expressed genes in liver tissue. Red represents upregulated genes, and blue represents downregulated genes **(B)**. Gee Oncology (GO) term enrichment analysis of differentially expressed genes **(C)**. KEGG pathway enrichment analysis of the first 20 differentially expressed genes obtained by Kyoto Encyclopedia of Genes and Genomes (KEGG) enrichment **(D)**.

A total of 21,090 genes were expressed in Groups C and H, with *p* < 0.05 and |log-2-fold change|> 1 as the threshold, and 1,375 DEGs were found. There were 794 upregulated genes and 581 downregulated genes in Group H when compared with Group C (Figure 2A). The DEG expression patterns of each sample were clustered on the basis of the log2 (fold change) values of their expression ratios, which exhibited good repeatability of samples in the two groups (Figure 2B).

To further elucidate the functional role of 1,375 DEGs, the GO and KEGG pathways were enriched to search for significant overabundance categories. GO terms were divided into three categories: biological processes, cell components, and molecular functions, and 47 terms (*p* < 0.05) were significantly enriched in the three categories. The first 30 terms were obtained through GO enrichment (that included 10 biological process terms, 8 molecular function terms, and 12 cellular component terms). Further analysis was performed to determine the relevant regulatory function (Figure 2C). Five terms were found to be related to lipid metabolism, including response to lipid (GO: 0033993), cellular response to lipid (GO: 0071396), response to steroid hormone (GO: 0048545), cellular response to steroid hormone stimulus (GO: 0071383), and steroid hormone-mediated signaling pathway (GO: 0043401). Five of these terms all belong to biological processes (Table 3).

**Table 3:** The significantly enriched terms associated with lipid metabolism.

**Term ID**	**Description**	***p*** **-value**	**Gene number**	**Gene name**
GO:0033993	response to lipid	0.035791763	6	*Rorc/Pparg/Nr3c2/Esrrg/Rxrg/Nr4a1*
GO:0043401	cellular response to lipid	0.035791763	6	*Rorc/Pparg/Nr3c2/Esrrg/Rxrg/Nr4a1*
GO:0048545	response to steroid hormone	0.035791763	6	*Rorc/Pparg/Nr3c2/Esrrg/Rxrg/Nr4a1*
GO:0071383	cellular response to steroid hormone stimulus	0.035791763	6	*Rorc/Pparg/Nr3c2/Esrrg/Rxrg/Nr4a1*
GO:0071396	steroid hormone mediated signaling pathway	0.035791763	6	*Rorc/Pparg/Nr3c2/Esrrg/Rxrg/Nr4a1*

A total of 1,375 DEGs were also integrated into the KEGG pathway database, and 32 pathways (*p* < 0.05) were significantly enriched. Figure 2D shows the first 20 significantly enriched pathways. Lipid metabolism and deposition involve five pathways, i.e., biosynthesis of unsaturated fatty acids pathway (mmu01040), steroid hormone biosynthesis (mmu00140) with 9 genes and 14 genes, 13 genes in peroxisome proliferator-activated receptors (PPAR) signaling pathway (mmu03320) and fatty acid elongation pathway (mmu00062) of 6 genes, tyrosine metabolism pathway (mmu00350) with 6 genes, and primary bile acid biosynthesis pathway (mmu00120) of 4 genes (Table 4). Combining GO and KEGG analysis, we screened 12 genes related to lipid metabolism (Table 5).

**Table 4:** The significantly enriched pathways associated with lipid metabolism.

**Pathway ID**	**Description**	***P*** **value**	**Gene number**	**Gene name**
mmu01040	Biosynthesis of unsaturated fatty acids	0.000321506	9	*Elovl3/Fads2/Acot4/Acot3/Scd1/Acot2/Acnat2/Gm40474/Acot5*
mmu00140	Steroid hormone biosynthesis	0.00033063	14	*Cyp17a1/Cyp2b9/Cyp2c54/Ugt2b1/Cyp2c70/Cyp2c37/Ugt1a9/Gm41857/Cyp2c38/Cyp2b13/Cyp7b1/Cyp2c39/Hsd3b5/Ugt2b38*
mmu03320	PPAR signaling pathway	0.001733084	13	*Ubc/Fads2/Cd36/Pparg/Fabp2/Cyp27a1/Pck2/Cyp4a31/Scd1/Rxrg/Cyp4a14/Cyp4a12b/Plin1*
mmu00062	Fatty acid elongation	0.014560226	6	*Elovl3/Acot4/Acot3/Acot2/Gm40474/Acot5*
mmu00350	Tyrosine metabolism	0.019064803	6	*Got1/Aox3/Ddc/Adh7/Adh6-ps1/Aldh3a1*
mmu00120	Primary bile acid biosynthesis	0.020084289	4	*Cyp27a1/Cyp46a1/Acnat2/Cyp7b1*

**Table 5:** Information of 12 differentially expressed genes associated with lipid metabolism.

**Gene name**	**Gene ID**	**log2FoldChange**	**C readcount**	**H readcount**	***P*** **-value**	**Descriptions**
*Pparg*	19016	1.49	108.59	304.67	2.31E−15	peroxisome proliferator activated receptor gamma
*Scd1*	20249	1.19	64,638.17	147,212.05	1.09E−08	stearoyl-Coenzyme A desaturase 1
*Rorc*	19885	1.31	400.22	990.88	2.20E−14	RAR-related orphan receptor gamma
*Fads2*	56473	1.03	8,223.41	16,791.25	1.24E−22	fatty acid desaturase 2
*Acot2*	171210	1.93	307.2	1,170.6	1.96E−08	acyl-CoA thioesterase 2
*Acot4*	171282	1.41	631.39	1,674.93	2.05E−16	acyl-CoA thioesterase 4
*Fabp2*	14079	1.19	88.6	167.69	2.66E−13	fatty acid binding protein 2
*Apoa4*	11808	4.08	5,675.48	95,974.87	7.93E−106	apolipoprotein A-IV
*Cyp46a1*	13116	1.63	24.96	77.45	9.04E−09	cytochrome P450, family 46, subfamily a, polypeptide 1
*Cyp27a1*	104086	−1.01	5,987.95	2,970.2	5.74E−12	cytochrome P450 family 27 subfamily A member 1
*Cyp7b1*	13123	−1.43	4,090.27	1,520.04	0.0006842	cytochrome P450, family 7, subfamily b, polypeptide 1
*Plin1*	103968	−3.39	4.16	0.42	0.0419683	perilipin 1
*Acot3*	171281	2.12	386.94	1,682.7	2.120216	Acyl-CoA Thioesterase 3
*Elovl3*	12686	−2.61	9,921.82	1,628.4	2.71E−89	ELOVL Fatty Acid Elongase 3

*Log2-foldchange of readcount by Group C (C readcount) vs. Group H (H readcount), of which 9 genes upregulated expression (log2-foldchange > 0) and 3 genes downregulated expression (log2-foldchange < 0)*.

### RT-QPCR Validation

**Figure 3:**
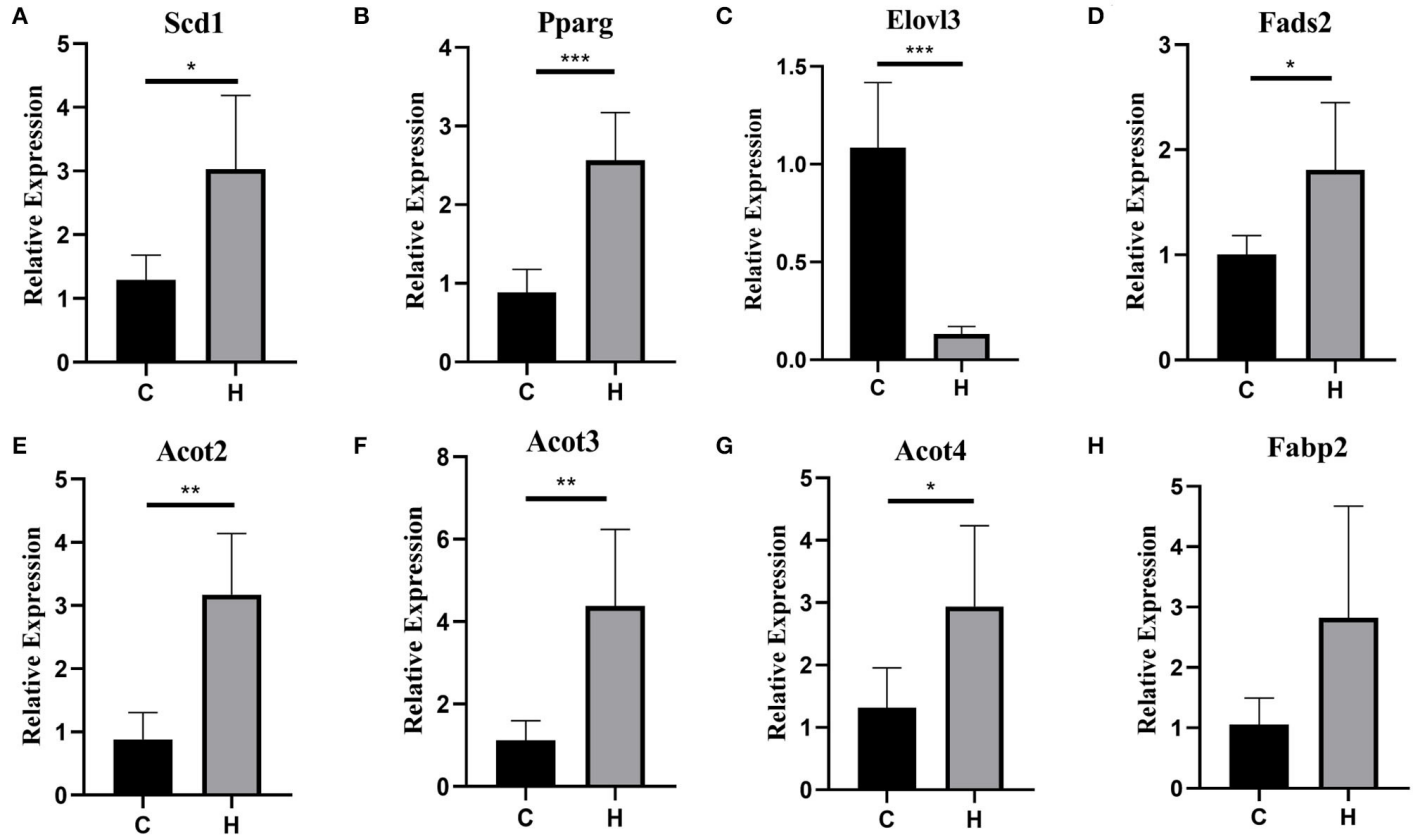
**(A–H)** Validation of 8 differentially expressed genes (DEGs) between the groups C and H mice by RT-qPCR. The x-axis denotes the group, and the y-axis denotes the log2-fold change derived from RT-qPCR. *p < 0.05, **p < 0.01, ***p < 0.001.

To verify the RNA-Seq expression results, we further determined the expression level of 8 lipid-related genes in the liver of mice from Groups C and H, i.e., Stearoyl-Coenzyme A desaturase 1 (*Scd1*), *Pparg*, ELOVL Fatty Acid Elongase 3 (*Elovl3*), fatty acid desaturase 2 (*Fads2*), acyl-CoA thioesterase 2 (*Acot2*), acyl-CoA thioesterase 3 (*Acot3*), acyl-CoA thioesterase 4 (*Acot4*), and fatty acid binding protein 2 (*Fabp2*), of the eight genes. Only one was downregulated, and all the others were upregulated, using RT-qPCR analysis. These 8 genes related to growth metabolism were selected from the KEGG pathways and the GO terms that were significantly enriched in relation to lipid metabolism. Interestingly, these DEGs were all upregulated. Although the expression levels of these 8 determined genes in the liver were higher in Group C than in Group H, *Fabp2* was not significantly different (*p* = 0.071), and *Scd1, Pparg, Elovl3, Fads2, Acot2, Acot3*, and *Acot4* were genes that showed a significant (*p* < 0.05) difference between Groups C and H (Figure 3).

### Differences in the Diversity and Composition of the Colon Microbiome in Mice

**Figure 4:**
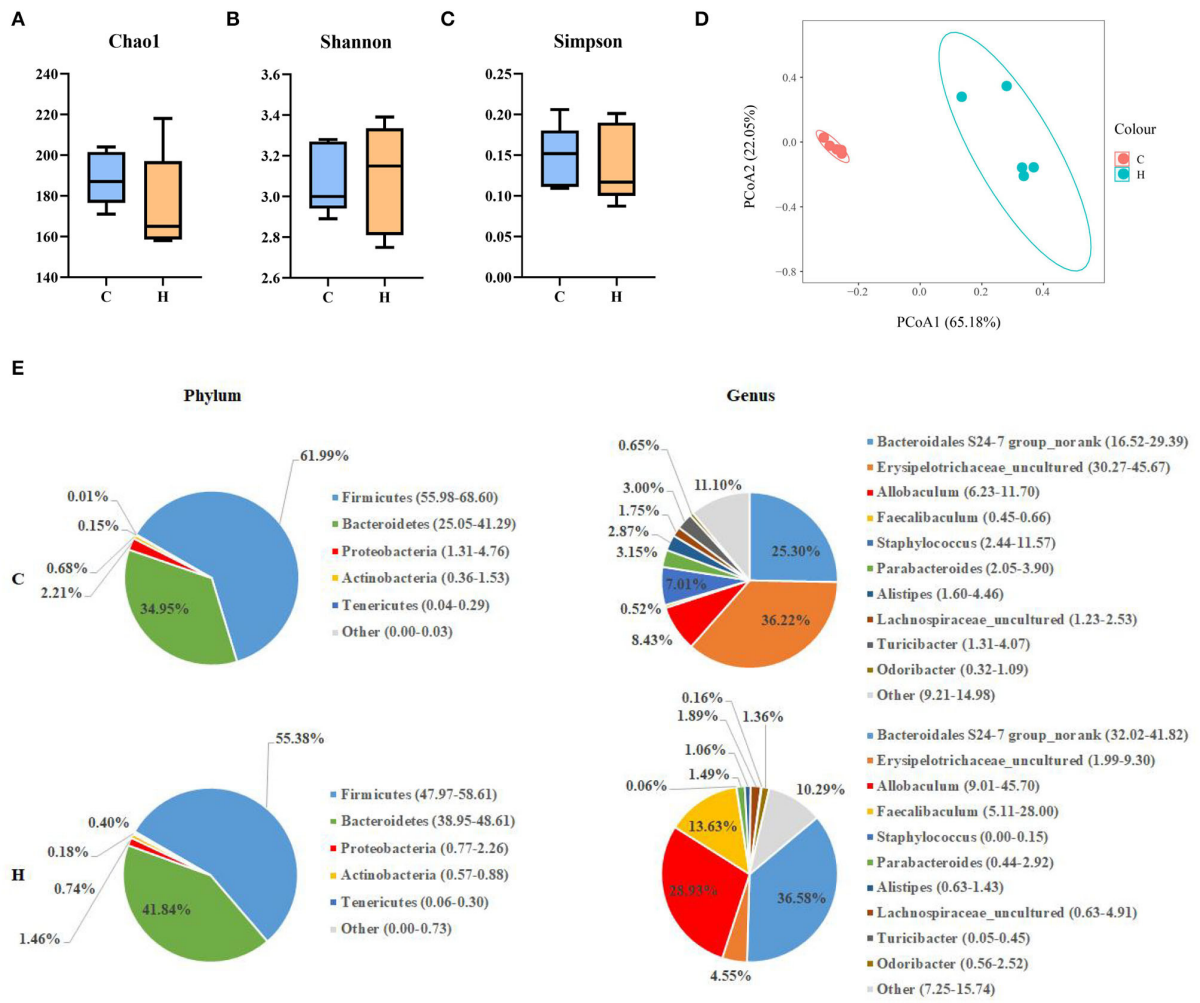
Alpha diversity, including the Chao1 index, Shannon index, and Simpson index **(A–C)**. Principal coordinates analysis (PCoA) of the difference in the microbial community composition of 10 colons in mice. The percentages present the relative contribution of the 2 principal coordinates (PC1–PC2) **(D)**; phylum- and genus-level pie charts of the microbial composition in the colon of Groups C and H mice **(E)**.

After 16SrRNA gene sequencing of colonic contents, the alpha diversity analysis structure indicated that Chao1 index, Shannon index and Simpson index indices were not significantly different (*P* > 0.05). It was shown that HFCS had no significant effect on the abundance of gut microbiota components in mice (Figures 4A–C). Beta diversity indicated that there was a large separation between Group C and Group H, indicating a considerable difference in gut microbial composition between the two groups (Figure 4D). To further investigate the effect of HFCS on intestinal microorganisms, we analyzed the microbial composition in the colon of mice in Groups C and H. Analysis of differences at the phylum level of the gut microbiome showed that when compared to Group C, the abundance of Proteobacteria and Actinobacteria was reduced in Group H. The abundance of Firmicutes was significantly reduced and Bacteroidetes was significantly increased in Group H. Analysis of differences at the genus level of the gut microbiome revealed that the abundances of *Allobaculum* and *Faecalibaculum* were significantly reduced, and the abundance of *Erysipelotrichaceae_uncultured, Parabacteroides*, and *Staphylococcus* was increased in Group H (Figure 4E).

### Linear Discriminant Analysis Effect Size (LEfSe) Identified Different Bacteria Between Groups C and H

**Figure 5:**
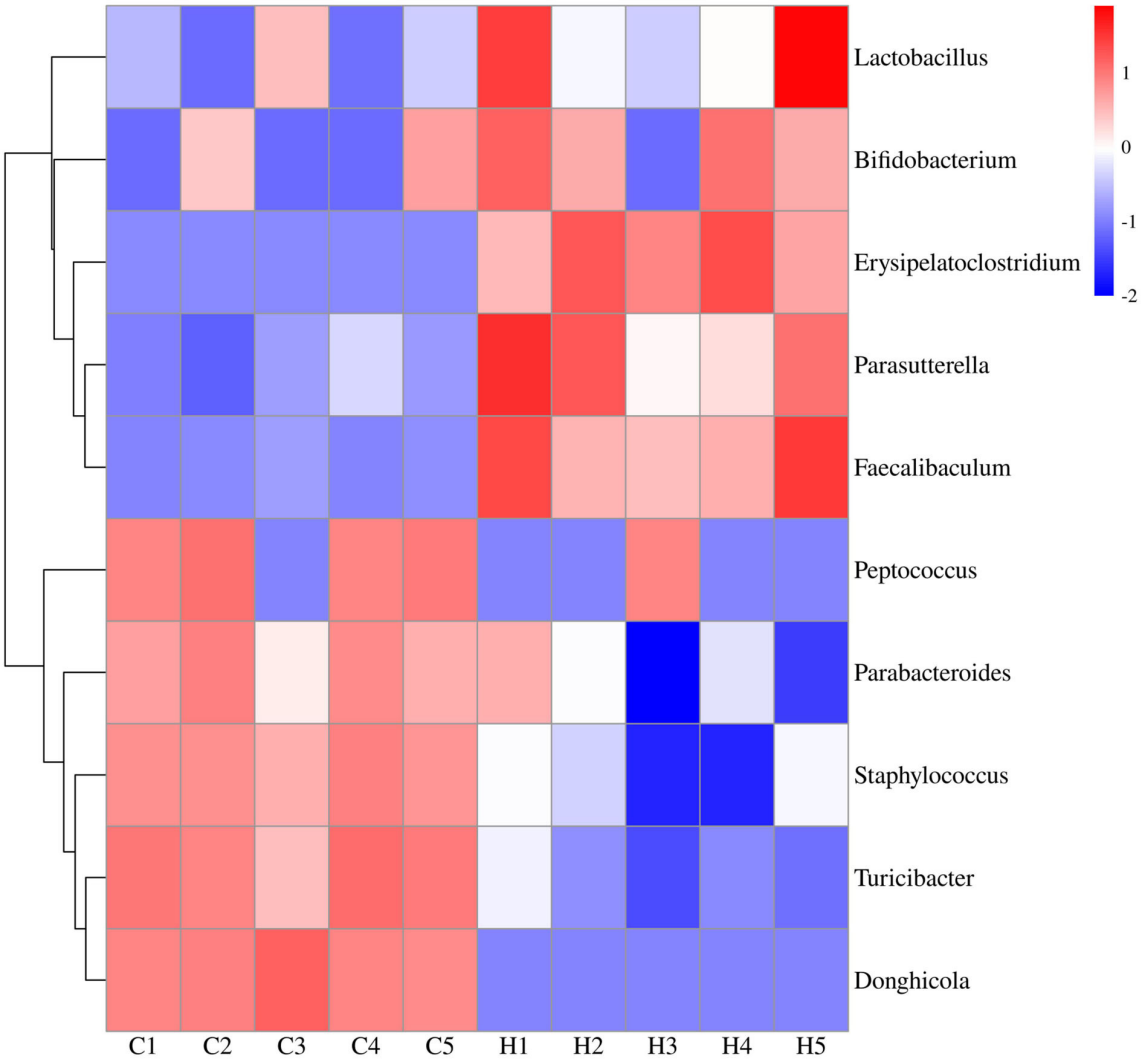
Expression profiles of 10 differentially expressed bacteria in colonic contents. The vertical axis represents the genus clusters, and one row represents one genus. The color scale represents the expression abundance of the genera; red represents increased abundance, and blue represents decreased abundance.

To further investigate the differences in the structure and abundance of mouse colonic bacteria at the level of 100 genera, heatmap analysis was performed on the differential genera obtained from LEfSe analysis. The results showed that there were obvious intergeneric differences in the colonic microbiome of mice. Under HFCS intervention, the relative abundance of *Bifidobacterium, Lactobacillus, Faecalibaculum, Erysipelatoclostridium*, and *Parasutterella* was increased in Group H, whereas the relative abundance of *Staphylococcus, Peptococcus, Parabacteroides, Donghicola*, and *Turicibacter* was reduced in Group H (Figure 5).

### Correlation Analysis of Differentially Abundant Genera and Lipid Metabolism-Related Genes in Mice

**Figure 6:**
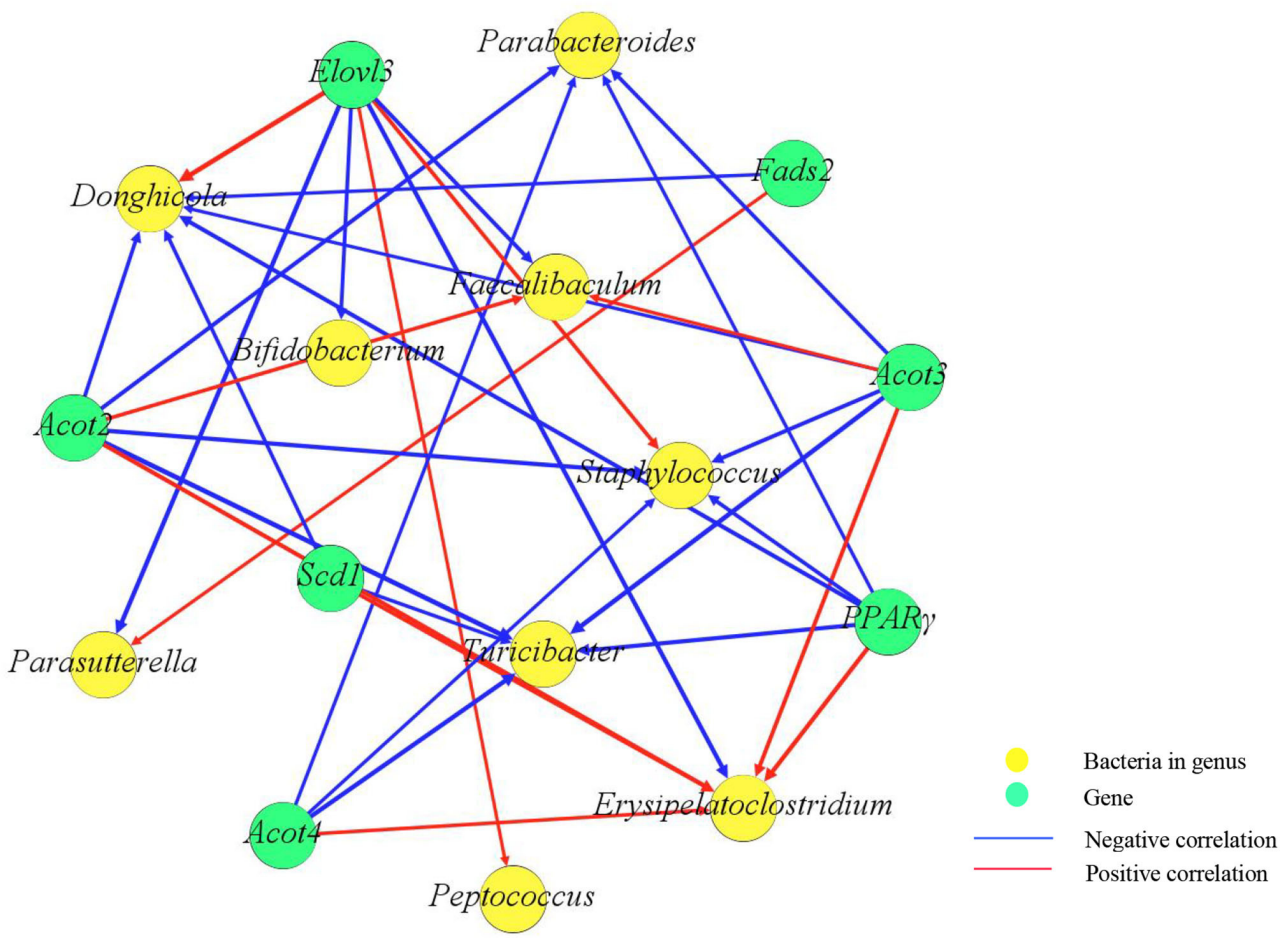
Correlation network diagram between genes and the gut microbiome. Yellow spots represent bacteria, and green spots represent genes. Red lines represent positive correlations, and blue lines represent negative correlations. The weight of the lines represents the strength of the correlation, and the thicker the line is, the greater the absolute value of the correlation. The number of spots represents the number of correlated objects.

Correlation analysis was performed to reveal the association between the gut microbiome and lipid metabolism-related genes. Through Spearman's correlation analysis, *Pparg, Acot2, Acot3*, and *Scd1* were positively correlated with *Erysipelatoclostridium* and negatively correlated with *Parabacteroides, Staphylococcus*, and *Turicibacter*. *Bifidobacterium* was negatively correlated with *Elovl3* (Figure 6).

## Discussion

High fructose corn syrup is a syrupy mixture of glucose and fructose. In recent years, HFCS has been widely used in the food industry and has mostly replaced sucrose with the advantages of low price and better taste. Bocarsly (28) showed that a short-term intake of 8% HFCS increased body weight, along with increased abdominal fat in mice. These results suggest that HFCSs may have profound negative impacts on the liver and adipose tissue. HFCS alters body metabolism (3), and we found that HFCS intake affects intestinal microbes in a previous study (29). In the present study, we found that HFCS intake resulted in a significant increase in body weight and epididymal and perirenal fat in mice (*p* < 0.001). Although there was no significant difference in liver weight, observation of liver sections revealed that the proportion of lipid droplet area in liver tissue was greater in Group H than in Group C. Collectively, we thoroughly investigated mouse colonic microbes by 16S rRNA gene sequencing and analyzed changes in the abundance and structure of mouse colonic microbes after HFCS intervention. Not surprisingly, various bacteria existed in the mouse colon, with the dominant phyla being Bacteroidetes and Firmicutes, giving a result similar to other studies. Furthermore, we identified a total of 1,375 DEGs in the two groups by transcriptome analysis. Compared with Group C, we found 794 upregulated and 581 downregulated genes in Group H and found pathways related to lipid metabolism and inflammation. In addition, RT-PCR results showed that the expression of the *Elovl3* gene was reduced, while the other genes, such as *Scd1, Pparg, Fads2, Acot2, Acot3, Acot4, and Fabp2*, were all upregulated in Group H. Finally, this study shows that HFCS induces abnormalities in hepatic lipid metabolism and alters the structural abundance of the gut microbiome.

The liver is an important metabolic organ in the body that plays a role in deoxidation, storage of liver sugar, and synthesis of secreted proteins (30). Past studies have shown that one of the causes of obesity is the accumulation of individual lipids, which leads to a series of diseases (31). HFCS is considered to be more lipogenic than sucrose, which increases the risk for non-alcoholic fatty liver disease (NAFLD) and dyslipidemia (32). Similarly, in the present study, we found that the administration of HFCS induced higher liver fat infiltration and the most extensive fat blisters as compared to controls.

To better understand the mechanism by which HFCSs regulate hepatic lipid accumulation, further transcriptome sequencing was performed. There were 794 upregulated and 581 downregulated genes between the two groups, which were enriched in lipid metabolic pathways. In particular, the most significant difference observed regards the “biosynthesis of unsaturated fatty acids,” which play multiple roles in human health (33). Functional analysis showed that the *Fabp2* and *Acot2* genes were related to liver regulation of lipid metabolism and IR through a series of biological processes (34). In the present study, the expression of *Acot2, Acot3*, and *Acot4* was significantly increased by HFCS treatment, suggesting that these three genes could promote adipocyte differentiation. *Elovl3* is known to promote fatty acid synthesis (lipogenesis), and RT-PCR results showed that the expression of the *Elovl3* gene was reduced. It was shown that the periodic expression of *Elovl3* in the liver may be controlled by the biological clock, related hormones, and transcription factors and confirmed that *Elovl3* has high and rhythmic expression in male mice (35). We speculate that the external environment may be a contributing factor in this phenomenon.

*Peroxisome proliferator activated receptor gamma* can control the peroxisome beta-oxidation pathway of fatty acids. Tontonoz et al. (36) reported that *Pparg* regulates adipocyte differentiation. The RT-PCR results showed that the expression of the *Pparg* gene was upregulated in Group H, and it is speculated that HFCS treatment increases the expression of *Pparg* and promotes the differentiation of adipocytes, which leads to the occurrence or aggravation of the individual obesity. Research shows that perilipin 1 (*Plin1*) expression is decreased in obese participants as compared to healthy controls (37). These findings demonstrate a novel role for *Plin* expression in adipose tissue metabolism and obesity regulation (38). *Fabp2* can help to maintain energy homeostasis by acting as a lipid sensor. *Fabp2* is a key factor in lipogenesis and lipid transport (34). In this study, we found that the *Fabp2* gene was enriched in the PPAR signaling pathway and that its expression was upregulated in Group H. Collectively, our findings suggested a functional role for *Pparg* and *Fabp2* in the regulation of HFCS-mediated hepatic lipid metabolism.

On the other hand, *Scd1* plays key roles in lipid storage, liposome homeostasis, and energy metabolism (39). The study of the *Scd1* gene mutant mouse strain provides evidence that *Scd1* is an important control point of lipid metabolism (40). Scd1-deficient mice had decreased liver fat content, suggesting the lack of *Scd1* protected mice from fat accumulation in the liver (41). This experiment revealed that the expression of *Scd1* was increased in the liver of mice by HFCS intervention, thus regulating the process of lipid metabolism, which is consistent with previous findings. In this study, Cytochrome P450 Family 27 Subfamily A Member 1 (*Cyp27a1*) and Cytochrome P450, Family 7, Subfamily b, Polypeptide 1 (*Cyp7b1*) were found to be reduced by HFCS intervention. Other studies have reported that Cyp27A1^−/−^ mice have an enlarged liver and kidneys and increased triglyceride levels and fatty acid synthesis, and cholesterol absorption and synthesis (42). Furthermore, *Cyp7b1* has broad substrate specificity, is widely distributed in most organs, and plays important roles in the regulation of lipid metabolism (43). Overall, alterations in gene expression levels can affect lipid metabolism.

Many studies have shown that the gut microbiome plays an important role in determining body weight (44). PCoA representation of the bacterial genera composition of all samples revealed that HFCS intervention altered the mouse colon microbiome when compared with Group C. Our expectation was confirmed that specific bacteria would exist in the mouse colon, with the dominant phyla being Bacteroidetes and Firmicutes, a result consistent with other studies (45). In the present study, mice in Group H had a lower relative abundance of the Firmicutes phylum and a higher relative abundance of the Bacteroidetes phylum than mice in Group C. This result was confirmed by Mastrocola et al. (19), who found that the ratio of Firmicutes/Bacteroidetes and abundance of Firmicutes were decreased in mice fed fructose (20), which is consistent with our findings. In the present study, the relative abundance of *Bifidobacterium, Lactobacillus, Faecalibaculum, Erysipelatoclostridium*, and *Parasutterella* was increased after the HFCS diet as compared to the normal diet, as shown by the genus level of the heatmap, in contrast to the decrease in the relative abundance of *Staphylococcus, Peptococcus, Parabacteroides, Donghicola*, and *Turicibacter. Lactobacillus* remained tightly attached in the intestinal epithelium (46), and the abundance of *Lactobacillus* was increased with sugar intake (20). *Bifidobacterium* was increased only in mice fed the Western-style diet (WSD) + fructose diet, which is consistent with our findings. *Lactobacillus* and *Bifidobacterium* are considered to have health benefits, but some studies have found harmful effects on physical health (47, 48). *Parabacteroides* have an anti-obesity effect, Julia Beisner et al. found that they decreased after a high-fructose diet (19). Wang B et al. observed changes in intestinal microorganisms in mice through different diets and found that some studies showed a lower abundance of *Turicibacter* and *Pediococcus* in fructose diets than in controls (49).

Some studies have shown that the gut microbiome affects energy metabolism by regulating key genes (50). Erysipelotrichaceae has a positive correlation with obesity (51), and *Turicibacter* has been closely associated with abnormal lipid metabolism (52). *Parabacteroides* have an anti-obesity effect (45). *Pparg, Acot2*, and *Acot3* can promote adipocyte differentiation. It has been shown that the knockdown of Scd1 inhibits adipogenesis (53). *Scd1* can play a vital role in lipid metabolism, and its product oleic acid (OA) can promote lipolysis by upregulating lipase (54, 55). In this study, we found that *Pparg, Acot2, Acot3, and Scd1* were positively correlated with *Erysipelatoclostridium* and negatively correlated with *Turicibacter* and *Parabacteroides. Bifidobacterium* can suppress fat accumulation and regulate obesity and related metabolic diseases by affecting lipid metabolism (56–58). Elovl3 deletion may lead to reduced lipid accumulation in the liver (59). Our results showed that *Bifidobacterium* was positively correlated with *Pparg* and *Acot3* and negatively correlated with *Elovl3*. Overall, gut microbes can regulate related genes to influence lipid metabolism.

## Conclusion

Our study demonstrated that HFCS intake resulted in a significant increase in body weight, epididymal and perirenal fat content, and an increase in the proportion of lipid droplet area in the liver, suggesting that HFCS may affect liver lipid metabolism in mice, leading to a lipid deposition. GO and KEGG analysis showed that *Pparg, Scd1*, RAR Related Orphan ReceptorC (*Rorc*), *Fads2, Acot2, Acot4, Fabp2*, apolipoprotein A-IV (*Apoa4*), cytochrome P450, family 46, subfamily a,polypeptide1 (*Cyp46a1*), *Cyp27a1, Cyp7b1*, and *Plin1*, 12 genes were screened and found to display keyregulatory roles in lipidmetabolism. The PCR results contributed to elucidating the metabolic effect of HFCS in reducing the expression of *Elovl3* and increasing the expression of *Scd1, Pparg, Fads2, Acot2, Acot3, Acot4, and Fabp2* in sthe liver. At the phylum level, the HFCS diet increased the abundance of the Firmicutes phylum while decreasing the abundance of the Bacteroidetes phylum. Obviously, the Firmicutes/Bacteroidetes ratio also was reduced. At the genus level, the relative abundance of *Bifidobacterium, Lactobacillus, Faecalibaculum, Erysipelatoclostridium*, and *Parasutterella* was increased; in contrast, the relative abundance of *Staphylococcus, Peptococcus, Parabacteroides, Donghicola*, and *Turicibacter* was reduced by the HFCS diet. Correlation analysis indicated that *Pparg, Acot2, Acot3, and Scd1* were positively correlated with *Erysipelatoclostridium* and negatively correlated with *Turicibacter*. We conclude that HFCS has a significant impact on genes related to lipid metabolism in the liver and changes the structure and function of the colonic microbiome in mice; in particular, it decreases the abundance of bacteria with anti-obesity effects, which suggests that it may lead to disruption of intestinal function and thus affect the body's metabolism. In general, HFCS regulates the expression of lipid metabolism genes in the liver by affecting the gut microbiome, thereby influencing organismal metabolism.

## Data Availability Statement

The data presented in the study are deposited in the NCBI (National Center for Biotechnology Information) repository, accession numbers: PRJNA731593 and PRJNA826752.

## Ethics Statement

The animal study was reviewed and approved by Zhejiang Provincial Ethics Committee for Laboratory Animals (Ethical Approval No.78865576).

## Author Contributions

YSh: methodology, review and editing, and data curation. YSu: methodology and review and editing. XW: methodology. YX: visualization. LM: supervision. WL: validation. ZZ: software. WW: funding acquisition, project administration, and resources. JL: conceptualization and investigation. All authors contributed to the article and approved the submitted version.

## Funding

This research was supported by the State Key Laboratory for Managing Biotic and Chemical Threats to the Quality and Safety of Agroproducts, Grant/Award Number: 2010DS700124-ZZ2017.

## Conflict of Interest

The authors declare that the research was conducted in the absence of any commercial or financial relationships that could be construed as a potential conflict of interest.

## Publisher's Note

All claims expressed in this article are solely those of the authors and do not necessarily represent those of their affiliated organizations, or those of the publisher, the editors and the reviewers. Any product that may be evaluated in this article, or claim that may be made by its manufacturer, is not guaranteed or endorsed by the publisher.
